# Telocytes of the male reproductive system: dynamic tissue organizers

**DOI:** 10.3389/fcell.2024.1444156

**Published:** 2024-10-14

**Authors:** Bruno D. A. Sanches, Lara C. Rocha, J. Pimentel Neto, Mateus Rodrigues Beguelini, Adriano P. Ciena, Hernandes F. Carvalho

**Affiliations:** ^1^ Department of Structural and Functional Biology, Institute of Biology, State University of Campinas (UNICAMP), Campinas, Brazil; ^2^ Laboratory of Morphology and Physical Activity (LAMAF), Institute of Biosciences, São Paulo State University (UNESP), Rio Claro, Brazil; ^3^ Center of Biological and Health Science, Federal University of Western Bahia (UFOB), Barreiras, Brazil

**Keywords:** telopodes, tissue organization, paracrine factors, stromal compartmentalization, prostate cancer, castration, testes, smooth muscle

## Abstract

Telocytes are CD34^+^ interstitial cells that have long cytoplasmic projections (called telopodes), and have been detected in several organs, including those of the male reproductive system. In this brief review we evaluate the role of telocytes in tissue organization of the different organs of the male reproductive system in which these cells were studied. In general terms, telocytes act in the tissue organization through networks of telopodes that separate the epithelia from the stroma, as well as dividing the stroma into different compartments. In addition to this contribution to the structural integrity, there is direct and indirect evidence that such “walls” formed by telocytes also compartmentalize paracrine factors that they or other cells produce, which have a direct impact on morphogenesis and the maintenance of organ cell differentiation, as well as on their normal physiology. Moreover, alterations in telocytes and telopode networks are correlated with pathological conditions in the male reproductive system, in response to profound changes in structural organization of the organs, in inflammation, hyperplasia and cancer. Further studies are necessary to evaluate the molecular pathways telocytes employ in different contexts of physiology and disease.

## Considerations on the morphology, distribution and function of telocytes

Telocytes are interstitial cells with long cytoplasmic projections, the telopodes (Popescu and Faussone-Pellegrini, 2010). These cells establish cellular junctions with each other and with other stromal and epithelial cells, enabling them to act as true connecting devices within different tissues (Cretoiu et al., 2019). Telocytes are distinct both morphologically and functionally from fibroblasts (Zheng et al., 2013; 2014), adding a new and interesting level to the complexity of the organization of different tissues. In comparison to fibroblasts, telocytes express a few more genes related to adhesion to the extracellular matrix, such as *Ctgf*, as well as genes related to changes in the cytoskeleton, such as *Tgln* and *Sprr1a*, in addition to genes related to mitochondrial transit, such as Myl9 (Zheng et al., 2013). Telocytes have more myosin-14 and periplakin, suggesting that telocytes might play specific roles in mechanical sensing and mechanochemical conversion, tissue homoeostasis and remodelling/renewal. Therefore, telocytes differ from fibroblasts both in having a more flexible and changeable cytoskeleton and in acting more actively in extracellular matrix remodeling.

In view of the diverse tissue-specific functions that have been proposed for telocytes, such as bladder contraction (Vannucchi et al., 2014; Traini et al., 2018; Neuhaus et al., 2020), immune function (Jiang et al., 2018; Huang et al., 2021; Aleksandrovych et al., 2022; Zhao et al., 2022), motility of the gastrointestinal tract (Banciu et al., 2022), nutrition of sperm in the testes (Marini et al., 2018), the regeneration of striated muscles (Manetti et al., 2019; Ravalli et al., 2021), myocardial contraction (Zhou et al., 2010; Mitrofanova et al., 2018; DeSimone et al., 2019) and many others. The general functions telocytes perform in different tissues include roles in cell-signaling, immune function, tissue-homeostasis, remodeling, and angiogenesis (Cretoiu et al., 2016). However, it is difficult to list among these functions which is the primordial or the first one that directed the evolution of these cells over time, since telocytes are found in vertebrates (Gandahi et al., 2020) and invertebrates (De Paula et al., 2022).

Telocytes possess Ca^2+^ reservoirs, therefore acting in calcium signaling and mainly at maintaining the mechanical resistance of the stroma (Edelstein and Smythies, 2014; Chaitow, 2017; Radu et al., 2017). This is a very basal function for telocytes and could exist even before the emergence of blood vessels and immune defense in metazoans.

Considering the broader aspect of the function of telocytes, in the organs of the male reproductive system and in other systems, evidence is accumulating that the very existence of the stroma is as dependent on telocytes as it is on fibroblasts. It means that the stroma of metazoans evolved through the association between telocytes, fibroblasts and other types of stromal cells, altogether correlated with the dynamics of epithelial cells. Therefore, it can be speculated that telocytes occupy regions abundant in cells and poor in collagenous extracellular matrix. As a matter of fact, telocytes showed three times higher ability to spread on some matrix proteins than fibroblasts. In these terms, telocytes adhere better to a fibronectin matrix, worse to a laminin matrix and intermediately to a collagenous matrix (Niculite et al., 2014). This is interesting because this adhesion preferences have a great impact on the organization of the stroma and may partially explain the complex arrangement of telocytes (Kubow et al., 2015). Interestingly, telocytes occupy regions with a looser matrix. This type of distribution in the stroma could explain why telocytes connect to distinct cell types, such as nerve cells, immune cells, endothelial cells, fibroblasts, and pericytes among others. The existence of their long, thin telopodes further aids these cells to set networks to connect different cell types, both mechanically and through paracrine signaling, during morphogenesis and homeostasis of different organs.

Therefore, unlike fibroblasts, which are embedded in the extracellular matrix they generate, telocytes are stromal cells that have evolved in close association with the cellular component of the stroma. This arrangement justifies the role attributed to telocytes of compartmentalizing different stromal components, such the layers of muscle, nerves, and blood and lymphatic vessels. However, telocytes have also evolved to occupy the periphery of the basal lamina of the epithelia, so that they end up acting to set barriers between the epithelium and the adjacent stroma. In these terms, telocytes can be understood as essential elements of the tissue organization of metazoans, which have accumulated several functions over evolutionary time, which justifies the multiplicity of roles that these cells can perform.

## Telocytes and tissue organization: general aspects

One of the few functions, if not the only one, that can be undisputedly ascribed to telocytes from the most varied tissues is a role in tissue organization. This premise was raised in early work by Popescu’s group, one of the researchers who coined the term telocyte, which indicated that telocytes play a role in the tissue organization of the myocardium (Bani et al., 2010; Bani, 2016). Since then, this hypothesis has also been raised for other organs (Sanches et al., 2017; Condrat et al., 2021). It must be considered that telocytes have homotypic junctions and that their processes, the telopodes, end up forming extensive networks that also connect with other cell types (Cretoiu and Popescu, 2014; Vannucchi, 2020; Cretoiu et al., 2020). Telocytes communicate with other cells mainly through direct junctions of their cytoplasm, the gap junctions, and through the exchange of extracellular vesicles. In the case of gap junctions, there is evidence for the exchange of small molecules and ions between telocytes and other cell types (Smythies and Edelstein, 2014; Popescu et al., 2015). There is evidence in several tissues that telocytes secrete extracellular vesicles (Cretoiu et al., 2016). Telocytes can absorb vesicles and exchange microRNA cargoes to cardiac stem cells (CSCs) (Cismaşiu and Popescu, 2015), to valvular interstitial cells (Yang et al., 2021), and to other cell types. There would also be the so-called stromal synapses that would connect telocytes with different cells, with a small gap between the membranes, leading to the suggestion that justacrine cell-to-cell signaling occurs (Popescu et al., 2005).

Telopode networks are present in the most diverse organs, and telocytes extend their telopodes to nerve endings, immune cells, epithelial cells, fibroblasts, and smooth muscle cells, among others, forming a complex network of interrelationships in terms of intercellular communication. At the same time, telocytes are also thought to act as true barriers capable of separating one tissue compartment from another. In this sense, it has been proposed that telocytes act in the compartmentalization of growth and differentiation factors, for example, in the prostate stroma (Corradi et al., 2013; Sanches et al., 2017), and in the maintenance of paracrine factors in the periphery of the developing prostate epithelium (Sanches et al., 2016). Simultaneously, telocytes also compartmentalize the stroma between the prostate alveoli and separate the prostate stroma from the periurethral smooth muscle. In this respect, studies that have been conducted over the last few years have brought us very promising insights into how effectively telocytes act in the organization of different tissues.

For didactic purposes, in this review, we will consider two ways in which telocytes act in tissue organization and we will emphasize the impact that they have on intercellular communication within tissues during development (Bani et al., 2010; Sanches et al., 2017), under normal physiological conditions (Banciu et al., 2016; Vannucchi, 2020; Rosa et al., 2021), and in pathological conditions (Manetti et al., 2014; Marini et al., 2018; Varga et al., 2019; Wishahi et al., 2023). Telocytes act directly on intercellular communication via the secretion of paracrine factors and extracellular vesicles (Cismaşiu and Popescu, 2015; Song et al., 2016; Cretoiu et al., 2016; Yang et al., 2021; Cucu et al., 2022; Sanches et al., 2024), being capable of modulating this communication in the three scenarios. At the same time, telocytes can also act as true physical barriers to compartmentalize paracrine factors secreted by other cells and, consequently, stimulate and/or maintain cellular differentiation in certain tissue compartments. In this sense, recent work carried out on the prostate using 3D reconstructions of SEM images (Kawaguchi et al., 2024) demonstrated that the telopodes and their dilations (podoms) can be flatter than assumed in 2D images; in fact, telocytes can act in certain contexts as, roughly speaking, true walls between one tissue compartment and another, which sheds light on how telocytes could separate different groups of paracrine factors and keep them restricted to a certain tissue region.

Telocytes are capable of regionalizing certain paracrine factors. In this respect, studies that were carried out on intestinal telocytes, with scRNAseq techniques performed on the different cells of the intestinal crypt, showed that telocytes produce different paracrine factors along their telopodes, so that in the vicinity of intestinal crypts that are rich in stem cells, they produce Wnt family members. In more distal regions of the villi, they produce factors from the Bmp family that stimulate the differentiation of intestinal stem cells into enterocytes (Shoshkes-Carmel et al., 2018; Kaestner, 2019). Therefore, telocytes act in the differentiation and tissue organization of the intestine in a spatially regulated manner (Kaestner, 2019). Furthermore, telocytes also interact with CD81^+^ fibroblasts that produce gremilin in the intestinal crypt and act in the maintenance of the stemness of intestinal stem cells (McCarthy et al., 2020). Additionally, telocytes in the hair follicle also act as a source of Wnts, supporting the idea that they function in the maintenance of the stem cell niche present in this compartment (Canella et al., 2023).

**FIGURE 1:**
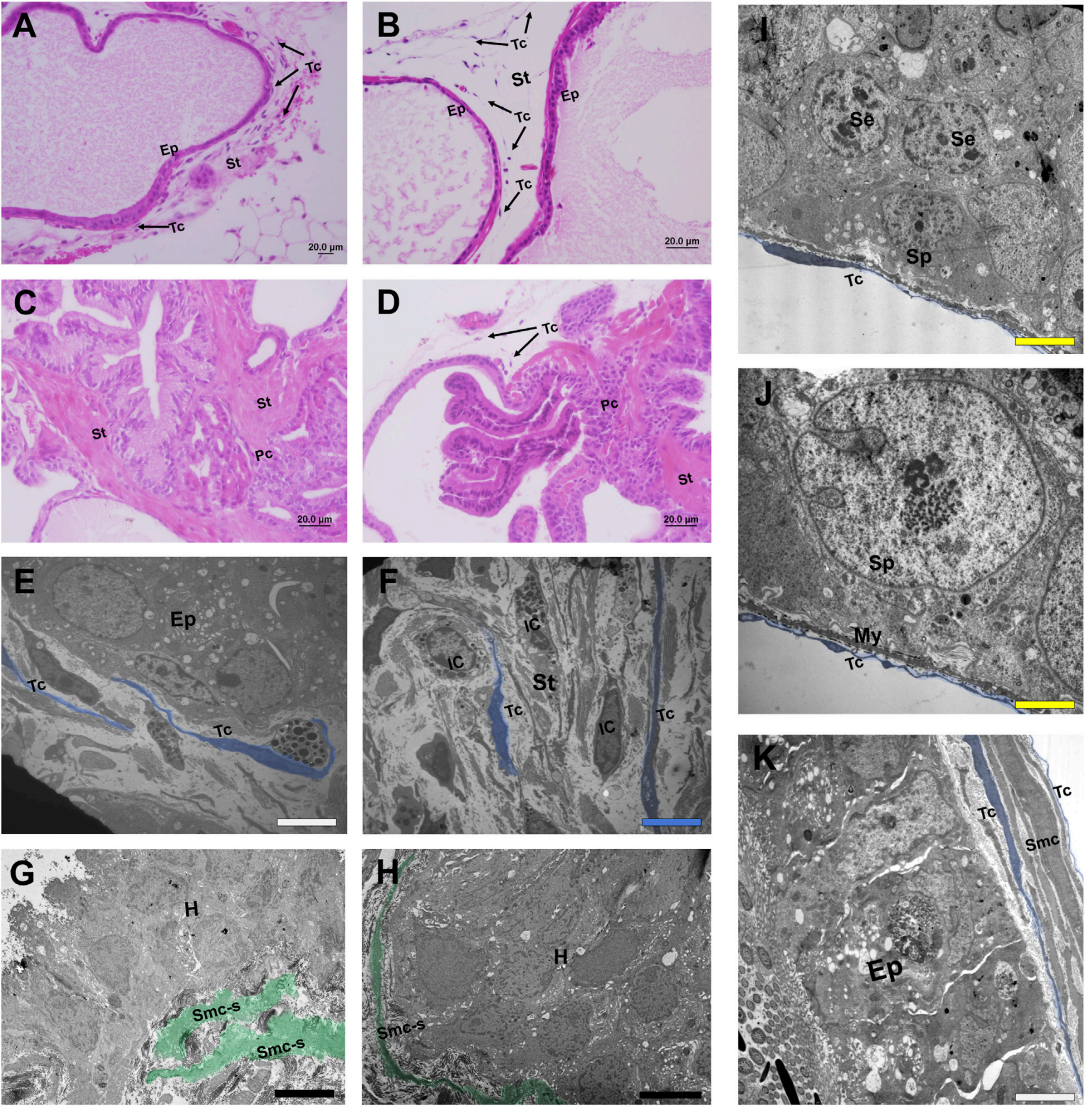
Histological sections stained in HE and micrographs representing telocytes of different organs of the male reproductive system. **(A, B)** Histological sections stained in HE from the normal prostate of a species of rodent (*Meriones unguiculatus*) in which telocytes are present both at the periphery of the prostate epithelium and in the interalveolar stroma. **(C, D)** In the case of prostate with adenocarcinoma foci, telocytes are located only in regions where the epithelium is intact. In the vicinity of an adenocarcinomous region, telocytes are absent and the stroma is repopulated mainly by myofibroblasts, losing its typical configuration. **(E)** Telocytes can be seen in the periepithelial region and interalveolar stroma in a TEM image of the rodent prostate (*Meriones unguiculatus*). **(F)** Telocytes spread their telopodes in the interalveolar stroma in the vicinity of immune cells in a TEM image of rodent prostate (*Meriones unguiculatus*). **(G, E)** Telocytes are absent around a focus of hyperplasia in a TEM image of the rodent prostate (*Meriones unguiculatus*); at the same time, it is possible to see smooth muscle cells that have switched their phenotype to the synthetic profile and layers of smooth muscles that are dissociated. **(I, J)** Telocytes present at the periphery of the epithelium of seminiferous tubules of the testes in close association with the myoid cells and in the vicinity of the spermatogonia in a TEM image of a species of bat (*Myotis nigricans*). **(K)** Telocytes in the vicinity of the epididymal duct and surrounding the epithelium and smooth muscle cells in a TEM image of a species of bat (*Platyrrhinus lineatus*). Ep (Epithelium), St (Stroma), Arrows, Tc, blue (Telocytes), Pc (Prostate cancer), IC (Immune cell), Smc-c, green (Smooth muscle cells transitioning to the synthetic profile), H (Hyperplasia foci), Sp (Spermatogonia), Se (Sertoli cells), My (Myoid cells), White bar (1 µm), Blue bar (2 µm), Black bar (5 µm), Yellow bar (0.5 µm).

We focus this review on the telocytes of the male reproductive system. This system encompasses both the production of male gametes and their survival, accumulation and transfer to the female reproductive tract (Swanson et al., 2001; Davies and Chapman, 2006; Dapper and Wade, 2020). It is noteworthy that telocytes have not been investigated in each organ of the male genital system, so this field of research remains open and promising (Sanches et al., 2021a; Gurung et al., 2024). Telocytes have been studied in most detail in the prostate and testes, and we will write about these cells in both organs and their role in tissue organization. We will also discuss aspects of the telocytes in less studied organs of the male reproductive system such as the seminal vesicle, urethra, vas deferens and epididymis (Figure 1).

## Telocytes in prostate tissue organization

The prostate is an accessory reproductive gland present in marsupial and placental mammals that acts in the survival and capacitation of spermatozoa (Verze et al., 2016). In histological terms, it is composed of an epithelium that is organized into ducts that open into the prostatic alveoli, which are the secretory unit of the gland. The prostatic epithelium is surrounded by a stroma that acts to provide mechanical support and nourishes it. This stroma contains smooth muscle cells that surround the ducts and alveoli, which contract to expel the prostatic secretion accumulated in the lumen of the alveoli (Ventura et al., 2002; Spek et al., 2021), as well as networks of blood vessels and nerve endings, in addition to fibroblasts, immune cells and telocytes. Telocytes are present in the prostate in the subepithelial region. Telopodes surrounds the basal lamina of prostate epithelial cells, as well as the perialveolar/periductal smooth muscles and spreading throughout the perialveolar region, establishing cellular junctions with other stromal cells; they also surround the prostatic stroma, separating it from the tissues of the adjacent organs (Corradi et al., 2013). Thus, telocytes have been proposed to act as a pivotal component of the prostate stromal organization.

Further studies are necessary to reveal the signaling pathways used by prostate telocytes in each of these contexts, as well as the different paracrine factors that these cells are capable of compartmentalizing in these different regions of the prostate. Recently, three-dimensional structural analysis using FIB–SEM tomography revealed sheet-like multilayered interstitial cells that appear to separate the prostate alveoli epithelium from the deeper interstitial tissue, which includes smooth muscle and capillaries (Kawaguchi et al., 2024). The cells that make up this structure that surrounds the alveoli were classified into three types, one that is exclusively PDGFRα+ and has a flat appearance, another that is PDGFRα+ and CD34^+^ that has a flat/sheet-like appearance and has a thick wall, constituting the periepithelial telocytes themselves, and a third that is oval and CD34^+^, which is possibly a telocyte progenitor cell. These recent findings show that telocytes, at least in the prostate, can have a flatter appearance in the 3D view and constitute a kind of irregular envelope around the prostate alveoli, which is consistent with the intimate relationship that telocytes have with the epithelium and smooth muscle cells previously described (Felisbino et al., 2019; Sanches et al., 2021b). Interestingly, it was recently verified that prostate telocytes also undergo phenotypic changes during cell division and exhibit shorter telopodes, as well as an increased cytoplasmic volume. Later they undergo cell division and, finally, the restitution of the telopodes (Sanches et al., 2023), which indicates that telocytes have a more dynamic morphology than previously thought.

## Telocytes in morphogenesis

Telocytes are present in a very early stage of prostate morphogenesis, involving the prostate buds as early as the seventh day of postnatal life, and as development progresses. These cells form networks of telopodes that compartmentalize clusters of prostate alveoli and also separate the periurethral smooth muscles from the rest of the prostatic stroma (Sanches et al., 2017). Telocytes influence the development of the prostate via paracrine pathways and play a role in the differentiation of periductal smooth muscles through the secretion of TGF_β1_. It has been shown that periductal smooth muscle differentiates when the growth of prostate branches is complete. In molecular terms, stromal cells, possibly the telocytes, secrete FGF10 early in prostate morphogenesis stimulating the proliferation of the prostatic epithelium and branching morphogenesis, and later, the production of TGFβ1 increases to simultaneously inhibit epithelial proliferation and stimulate smooth muscle differentiation (Prins and Putz, 2008; Thompson-Elliott et al., 2021). It is noteworthy that telocytes are also sensitive to estradiol, expressing one of its receptors (ERβ) (Sanches et al., 2016), and that stromal estrogen receptors play an important role in early prostate development.

Moreover, there is evidence that prostatic stromal cells surrounding the developing epithelium secrete Wnts in a spatially regulated manner (Wei et al., 2019) so that the most proximal portions of the ducts would secrete more Wnts, activating pathways for the maintenance of prostate epithelial stem cell populations and inhibiting the their proliferation (Wei et al., 2019). It has long been known that Wnts inhibit the proliferation and differentiation of the prostate epithelium (Prins and Putz, 2008). Given the position that telocytes occupy in the prostate, it is possible that telocytes are these Wnt reservoirs. This has already been verified in other organs such as the intestine (Shoshkes-Carmel et al., 2018) and the endometrium (Chen et al., 2023).

## Telocytes rearrangements after castration, during aging and in pathological conditions

An interesting feature of prostate telocytes is their sensitivity to androgens, just like the gland itself. Experiments on castrated rodents demonstrate that the hypoandrogenic environment leads to the disaggregation of the telopode networks around the alveoli and makes the telocytes atrophic; simultaneously the gland epithelium regresses and the perialveolar/periductal smooth muscles become disorganized. However, after testosterone replacement, telocytes expand their telopodes again and the perialveolar/periductal smooth muscles reorganize (Felisbino et al., 2019). Therefore, it has been argued that telocyte networks are essential for both the maintenance of smooth muscles and the prostate epithelium (Sanches et al., 2021b), with the prostate telocytes forming a functional unit that is sensitive to steroid hormones together with smooth muscle cells. This is particularly interesting given the fact that many species that reproduce seasonally present an involution of the prostate in the non-reproductive period followed by an expansion of the gland in the reproductive phase (Zhang et al., 2022; Ishiguro et al., 2023). Telocytes seems to be a key factor in the reestablishment of the prostate gland functional architecture in the reproductive season.

In the aging prostate telopode networks support the structural integrity of the prostate alveoli as they dilate and the perialveolar muscles stretch. However, telocytes also secrete VEGF, a pro-angiogenic factor; concomitantly they are positive for TNFR1 and may also act as pro-inflammatory cells (Sanches et al., 2020). In terms of pathological conditions, telocytes and telopode networks are absent in the periphery of benign lesions, such as foci of hyperplasia, or pre-malignant, as in the surroundings of PIN, implying that telocytes are important for both the maintenance of smooth muscle and prostatic epithelium integrity, which indicates a protective role against the advancement of tumorigenesis. Telocytes are also closely related to inflammatory cell infiltrates present in the prostate, which suggests a complex role of telocytes in prostatitis (Maldarine et al., 2022).

Furthermore, telocytes could also contribute to the origin of cancer-associated fibroblasts. In cases of prostate cancer, CD34^+^ fibroblasts are recruited into tumor-associated reactive stroma, and these cells would contribute to cancer progression (San Martin et al., 2014). Telocytes can contribute to the progression of tumorigenesis via the production of TGFβ1, since this factor stimulates the fibroblast to myofibroblast transition (Midgley et al., 2013; Owen et al., 2022). In as much, the hypothesis that telocytes are capable of differentiating into myofibroblasts has already been considered for other organs previously (Vannucchi et al., 2016). However, the role of TGFβ1 is more complex, considering that this factor is also relevant for maintaining differentiated smooth muscle cells (Cao et al., 2022). Thereby, telocytes would contribute to maintaining the integrity of the prostatic stroma.

In fibrosis, there is excess deposition of ECM components, primarily the fibrillar collagens (Wight and Potter-Perigo, 2011; Antar et al., 2023) and it is interesting that the telopod networks of telocytes are discontinuous or absent, and the telocytes are reduced in number (Manetti et al., 2014; Wei et al., 2022). Therefore, it can be speculated that a collagen-rich matrix implies in the reduction of the number of focal adhesions and the matrix typical of fibrosis hinders the adhesion of telocytes and the spreading of their telopods. It is interesting to note that in a hypoandrogenic environment, prostatic telocytes dissociate from the smooth muscle layer, which undergoes the transition to a synthetic profile, and that the telocytes present phenotypic changes compatible with the synthetic profile. It is interesting that the phenotypic change of smooth muscle cells from the contractile to the synthetic profile involves the production of FGFs, which inhibit the action of TGFβ1 in maintaining the expression of contractile factors in these cells (Cao et al., 2022). Thus, the role of telocytes in maintaining periductal smooth muscle in the prostate possibly involves a regulated production of TGFβ1 and a reduced synthesis of FGFs.

During development, stromal cells, possibly telocytes, have been attributed as sources of FGFs that would lead to epithelial proliferation and prostate branching, but this production decreases as TGFβ1 production increases and the epithelium differentiates along with the periductal smooth muscle layers (Prins and Putz, 2008). It is interesting that FGFs are related to the progression of prostate cancer (Memarzadeh et al., 2007; Teishima et al., 2019), as well as the Wnts pathway (Wang et al., 2008; Wang et al., 2021) and other factors that are very active during early prostate development. In these terms, it has been proposed that prostate cancer reactivates many of the genetic programs related to prostate development (Marker, 2008). More recently, there is also evidence that epigenetic programs are also reactivated on a large scale in the progression of prostate cancer (Pomerantz et al., 2020).

## Telocytes in the tissue organization of the testis

The production of sperm and androgens is the main function of the testis. This depends on both testicular somatic cells and germ cells (Mäkelä et al., 2019). The testes are histologically composed of seminiferous tubules, in which spermatogenesis takes place, and an intertubular stroma that is composed of blood vessels, myoid cells, and telocytes. Telocytes form a distinct layer that surrounds the seminiferous tubules along with the inner layer of peritubular myoid cells. Within the testicular interstitium, telocytes form a network connecting peritubular myoid and Leydig cells as well as blood vessels and it was verified that testicular telocytes express CD34 and PDGFRα (Liu et al., 2019). Due to the arrangement of telocytes within the testes, it was proposed that they could provide structural support for the seminiferous tubules and that these cells are components of the blood–testis barrier that control the transfer of molecules and cells from the interstitial bloodstream to the germinal compartment of seminiferous tubules, thus being potentially involved in the regulation of spermatogenesis (Marini et al., 2018). Interestingly, telocytes have been detected in the testes of the Chinese soft shelled turtle *Pelodiscus sinensis* and, similarly to what is seen in other species, telocytes are also connected with each other and they also have intercellular junctions with myoid and Leydig cells in addition to blood vessels (Yang et al., 2015).

In terms of the role of telocytes in pathological conditions, in the testes of mice with Duchenne Muscular Dystrophy (DMD), Leydig cells were hypertrophic and the intertubular volume increased, but telocytes were unchanged (Braz et al., 2022), which indicates that telocytes can act to maintain the integrity of the stroma of the testes affected by this dystrophy. In the same vein, in cases of seminoma, which is a tumor that affects the germ cells and leads to the loss of the tissue organization of the testes, the telocytes and their telopode networks are absent, which corroborates that telocytes are important for the maintenance of testicular tissue organization (Marini et al., 2018). Interestingly, the intimate connection between telocytes and myoid cells in the testis resembles that seen in the prostate, since prostate telocytes surround the periductal/perialveolar muscles (Corradi et al., 2013). However, future studies are necessary to evaluate the role of telocytes in testicular morphogenesis, as well as the nature of the paracrine signaling that they might establish with Leydig and myoid cells and testicular blood vessels; such studies are necessary for a more mechanistic understanding of the role played by telocytes in the integrity and tissue organization of the testes.

## Telocytes in other organs of the male reproductive system

### Seminal vesicle

The seminal vesicle is a male reproductive accessory gland. It produces a secretion that contributes to sperm motility, capacitation and survival (Noda and Ikawa, 2019). In histological terms, the seminal vesicle is composed of a highly folded mucosa, which is divided into the secretory epithelium and a poorly developed lamina propria of connective tissue. Underneath the mucosa, there is a thick smooth muscle layer that contracts during ejaculation and expels secretion from the seminal vesicle into the ejaculatory ducts. In the seminal vesicle, telocytes and their telopodes were interconnected by homo- and heterocellular junctions and form a complex network between different cell types. Telopodes exhibit close contact with immune and progenitor stem cells, in addition to smooth muscle and other interstitial cells. Interestingly, telocytes in the seminal vesicle are sensitive to melatonin, which leads to an increased number of these cells and promotes their secretory activity (Abd-Elhafeez et al., 2017). Another study detected PDGFRα+ interstitial cells in the periepithelial region of the seminal vesicle that correspond to periepithelial telocytes, and it was found that they develop synchronous Ca^2+^ oscillations and electrical slow waves in a concatenated manner with the gland smooth muscle cells. Thus, it was argued that telocytes could perform the function of pacemaker cells to drive the spontaneous contractions of the seminal vesicle smooth muscles (Takeya et al., 2022). In general terms, new studies are necessary to investigate the impact of telocytes on the tissue organization of the seminal vesicle, especially during development and in pathological conditions. It remains to be determined what paracrine factors telocytes use in the interplay with smooth muscle and epithelial cells of the seminal vesicle.

### Urethra

The urethra is a dynamic fibromuscular tube that serves as the terminal region of both the male urinary and reproductive systems. The average male urethra is 20 cm long and begins within the bladder wall and ends in the distal glans of the penis. Overall, the function of the male urethra is to allow passage of urine and semen. In histological terms it is composed of an epithelium lining the lumen, which is adapted to deal with urine and semen. Surrounding the epithelium is the submucosal layer, which is quite vascularized and provides support to the epithelium. This layer is encircled by a fibromuscular layer that provides structure, propulsion, and tone to the urethra (Stoddard and Leslie, 2023). The urethral telocytes are concentrated in the subepithelial region and their telopodes form a network. In addition, telocytes are also found around blood vessels and nerve endings. It was also found that some of the urethral telocytes express ER and PR, which indicates that they are responsive to steroid hormones (Gevaert et al., 2012). In summary, urethral telocytes may play a role in tissue organization and in the cellular communication that occurs between the epithelium and the urethral stroma. Nevertheless, further studies are necessary to reveal the molecular factors underlying this communication, as well as to evaluate the role of telocytes in urethral morphogenesis and pathological conditions.

### Vas deferens

The vas (or ductus) deferens is a thick-walled muscular tube whose function is to convey spermatozoa from the epididymis to the urethra. The vas deferens secretory epithelium contribute to the medium in which the spermatozoa are bathed during transit. In histological terms, the wall of the vas deferens is composed of a mucosa, which comprises the vas deferens epithelium and a supporting elastic connective tissue, a muscle coat (the muscularis), and an adventitia. The muscle coat contracts and is important for sperm transport, and it is surrounded by a layer of loose connective tissue (the adventitia) that contains numerous nerve bundles and large blood vessels (Dixon et al., 1998). In a recent study, it was found that PDGFRα-positive cells were distributed in the lamina propria, smooth muscles, and serosal layers; some of these cells were CD34^+^ and were thus telocytes (Hiroshige et al., 2021). Telocytes form networks of telopodes that spread throughout the histological compartments of the vas deferens and can play an important role in intercellular signaling via direct contact with other stromal cells or through the release of extracellular vesicles.

### Epididymis

The epididymis is an essential reproductive organ responsible for sperm concentration, maturation (including sperm motility acquisition and fertilizing ability), protection and storage. It is a duct-like organ that connects the testis to the vas deferens, and sperm maturation occurs during epididymal transit by the interaction of sperm cells with the unique luminal environment typical of each epididymal region (James et al., 2020). In histological terms, the epididymis is composed of the epididymal duct, which has a coiled configuration. Around this duct is the peritubular stroma, which is composed of fibroblasts, smooth muscle cells, blood vessels and, discovered recently, telocytes. Two types of telocyte have been detected in the peritubular stroma; one type is distributed around the capillaries and has full cell bodies, long telopodes and many secretory vesicles; the other is distributed outside the basement membrane with irregularly long, striped, large nuclei and short telopodes. These telocytes form networks of telopodes that connect to epididymal interstitial capillaries and basal fibroblasts (Yang et al., 2023). In a similar study carried out on mice, telocyte cells (CD34+/PDGFRα+) were detected in the interstitial space and were associated with the contraction of the epididymal muscles, along with other PDGFRα+ interstitial cells that were detected well beneath the epithelium. In any case, the telocytes that were detected established connections between themselves and with nerves and macrophages (Hiroshige et al., 2021) and are an important components in the tissue organization of the epididymis.

### Final considerations and future directions

**FIGURE 2:**
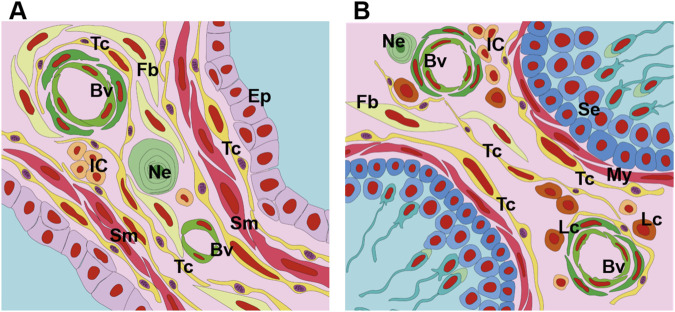
Schematic drawing depicting the telocytes of the prostate and testes. **(A)** Telocytes are present in the prostate both in the periepithelial region and in the interalveolar stroma. These cells form networks of thin processes (telopods) that connect them to each other and also to other cell types. In the prostate, telocytes are in close association with epithelial cells, muscle cells, nerve endings, blood vessels, immune cells and fibroblasts. **(B)** In the testes, telocytes are associated with the myoid cells that surround the seminiferous tubules; these cells also occupy the intertubular stroma and their telopode networks are linked to blood vessels, Leydig cells, fibroblasts, immune cells and nerve endings. Tc, yellow (Telocytes), Ep (Epithelium), Sm (Smooth muscle cell), Fb (Fibroblast), Ne (Nerve ending), My (Myoid cell), Se (Sertoli cell), Lc (Leydig cell), IC (Immune cells).

There is direct and indirect evidence of the role of telocytes in the tissue organization of organs of the male reproductive system. Such cells have been detected in the prostate and testes, in which there have been the most in-depth studies. There have also been studies that have analysed the presence of telocytes in the seminal vesicle, the urethra, the vas deferens and the epididymis. In all these organs, it has been found that telocytes form networks of telopodes that can be divided into those that are concentrated on the periphery of the epithelial cells, in addition to those that are interspersed in the stroma and involve the smooth muscle layers or spread through them, as well as extending through the connective tissue of these organs, being on the periphery of fibroblasts, blood vessels, nerve bundles and resident immune cells (Figure 2). Regarding the role of telocytes in tissue organization, it must be noted that they can secrete paracrine factors, as seen in prostate morphogenesis, and are capable of compartmentalizing paracrine factors secreted by other cell types, thus creating different molecular microenvironments. Interestingly, prostate telocytes can have a flattened morphology capable of forming sheet-like structures that contribute to this abovementioned compartmentalization of paracrine factors. Among the indirect evidence of the importance of telocytes in the tissue organization of the male reproductive system organs, telopode networks are absent in the case of seminoma in the testes, and in the periphery of benign and pre-malignant lesions in the prostate, and in the castration scenario. Such studies show that the changes and/or absence of telopode networks occur together with regression of the epithelia, as well as the smooth muscle layers and changes in the composition of the connective tissues.

The presence of telopode networks on the periphery of the epithelia of adult organs of the male reproductive system may imply the maintenance of the normal physiology of these organs, with potential impact on reproductive capacity. Considering that telocytes in the testes form a network that surrounds myoid cells, it has been suggested that they can provide structural support to the seminiferous tubules and that these cells are components of the blood–testis barrier, and that they could also act to transfer molecular elements from the interstitial bloodstream to the germinal compartment of seminiferous tubules. In the prostate, such cells may have a similar function in mediating epithelial–stromal interactions, as in the distribution of steroid hormones from the bloodstream to the epithelium. But a more general function for telocytes in the organs of the male reproductive system lies in their intimate association with layers of smooth muscle cells, so that the former can either surround such layers or be interspersed within them, indicating possible roles in maintaining the normal physiology and the differentiation of the smooth muscle cells in the male reproductive tract. This aspect of telocyte physiology is evident in the prostate remodeling after androgen deprivation, in which the telopode networks are lost and the telocytes display an atrophic profile; at the same time, the smooth muscle layers disaggregate, and smooth muscle cells undergo a phenotypic transition to the synthetic profile.

In general, the role of telocytes in the reproductive system involves possible support in the contraction of the smooth muscle layers of the organs, as proposed for the accessory glands, such as the prostate and the seminal vesicle. In the case of the seminal vesicle, it has been shown that telocytes develop synchronous Ca^2+^ oscillations and electrical slow waves along with the smooth muscle cells, which indicates a pacemaker role played by telocytes. In addition to these glands, the smooth muscles involve the tubular organs of the male reproductive system such as the epididymis, vas deferens and urethra. Therefore, telocytes may also be implied in the transit of sperm through the male reproductive tract.

Regarding the search for new therapeutic targets, the male reproductive system, SDF-1 has been shown to stimulate the proliferation of telocytes from the prostate gland *in vitro*, and emerges as a therapeutic target to increase the telocyte population in pathological conditions in which the networks of these cells are lost, such as in fibrosis and cancer. Another future direction in terms of therapy is the use of extracellular vesicles directly targeting telocytes with cargoes aimed at stimulating the proliferation of these cells, such as SDF-1. Another important future direction for the therapeutic use of telocytes is the establishment of commercial lines and the standardization of their genetic markers (Sanches et al., 2024).

In conclusion, telocytes act in the tissue organization of the organs of the male reproductive system through the networks of telopodes that separate the epithelia from the stroma of these organs, as well as dividing the stroma into different compartments. In addition to this structural role of maintaining the integrity of the organs, there is direct and indirect evidence that such “walls” formed by telocytes also compartmentalize paracrine factors that they or other cells produce. This observation is compatible with the impact of telocytes both during morphogenesis and the maintenance of cell differentiation, as well as the normal physiology of these organs. Finally, alterations in telocytes and telopode networks are correlated with pathological conditions in the male reproductive system in the contexts of hyperplasia and cancer. However, further studies are necessary to unveil the molecular pathways used by telocytes during development, under normal physiological conditions and in pathological conditions, which makes the study of telocytes in the male reproductive system a broad field for research.

## Abbreviation

CD34, Cluster of Differentiation 34; SEM, Scanning Electron Microscope; scRNAseq, Single-cell RNASeq; nt, Wingless-related Integration Site; Dkk3, Dickkopf-related Protein 3; sFRP1, Secreted Frizzled-related Protein 1; Bmp, Bone Morphogenetic Protein; CD81, Cluster of Differentiation 81; FIB–SEM, Focused Ion Beam-scanning Electron Microscopy; PDGFRα, Platelet-Derived Growth Factor Receptor α; TGFβ1, Transforming Growth Factor β 1; ERβ, Estrogen Receptor β; VEGF, Vascular Endothelial Growth Factor; TNFR1, Tumor Necrosis Factor Receptor 1; PIN, Prostatic Intraepithelial Neoplasia; TCs, Telocytes; DMD, Duchenne Muscular Dystrophy; Tps, Telopodes; Ca2+, Calcium; ER, Estrogen Receptor; PR, Progesterone Receptor; ICs, Interstitial Cells.
